# Irreversibility and Quantum Measurement

**DOI:** 10.3390/e28091019

**Published:** 2026-09-12

**Authors:** Julio Gea-Banacloche

**Affiliations:** Department of Physics, University of Arkansas, Fayetteville, AR 72701, USA; jgeabana@uark.edu

**Keywords:** wavefunction collapse, open quantum systems, epistemic interpretation, decoherence

## Abstract

The role played by irreversible processes, at the microscopic scale, in several examples of quantum measurements is studied within a “minimally realist” interpretation of the quantum formalism introduced in a recent publication. The analysis extends and confirms the basic results of the earlier work: separation of the collapse and amplification phases of the measurement, and decoherence and collapse happening already as a consequence of irreversible microscopic processes at the level of the “probe” itself (typically an atom in the examples considered). Additionally, the proposed interpretation of the formalism is shown to be consistent with previous interpretations, and a case for true irreversibility in physics is argued in some detail.

## 1. Introduction

The “measurement problem,” generally speaking, refers to the impossibility of deriving all or part of the measurement postulate of quantum mechanics by treating both the system to be measured and the measurement apparatus (plus as much of their environment as necessary) as a closed system evolving in accordance with the Schrödinger equation. This impossibility has perhaps been expressed most forcefully in J. S. Bell’s “Against Measurement” paper [1].

Historically, the main motivation for attempting such a derivation has stemmed from a desire to understand the so-called “collapse of the wavefunction” [2], and hence, it is probably felt most strongly by those who favor an “ontic” interpretation of the quantum formalism; that is to say, those who regard the wavefunction, or more generally the “state vector,” as somehow “physically real.” Since it is mathematically impossible to derive the collapse (where information about the initial state disappears) from the information-conserving Schrödinger equation, supporters of the ontic interpretation either deny the collapse altogether (many-worlds interpretation) [3], propose modifications to the Schrödinger dynamics [4,5], or postulate a limit to the applicability of the wavefunction concept, whether this stems from the size of the system or the nature of the processes involved (see, e.g., [6,7], and references therein).

The difficulty is felt less strongly by those who favor (implicitly or explicitly) an “epistemic” interpretation, which takes the wavefunction to be, ultimately, just a mathematical repository of the information we have about the system, and its “collapse” as merely our updating of that information (see, e.g., [8]). One can then take the position that it is not the Schrödinger equation’s job to predict, much less describe, the change that presumably happens in the “real world” as a result of the measurement: instead, measurement can be taken to be, as the founders wanted it, a “primitive” of the theory, not explained by it, and the wavefunction, or “state vector,” merely a tool we use to calculate the probability of the various outcomes (which, of course, it does correctly). A popular interpretation of quantum mechanics along these lines, QBism [9], explicitly makes this point when it denies, in the words of D. Mermin [10], “that there are properties of the external world—“elements of reality”—that underlie quantum state assignments.”

Nevertheless, although an epistemic interpretation of the formalism makes such a position logically unassailable, it does not actually mandate it, and there is, in fact, reason to believe that it was not shared by at least some of the founders (particularly Heisenberg, as I will argue below). In a previous paper [11], I have proposed instead a “minimally realist” epistemic interpretation that allows for the possibility that under certain conditions our state assignments may in fact, reflect external elements of reality. It was shown there that, under such an interpretation, a consistent description of a quantum measurement is possible, provided the coupling of the probe to the environment is such as to guarantee the irreversibility of the process. Interestingly, in the example considered there, the state reduction process could be seen to happen already at the microscopic level, and was conceptually separate from the subsequent amplification stage. This led to the central claim of [11], namely, that microscopic irreversibility, rather than macroscopic amplification, is sufficient to legitimize the replacement of a pure-state by an incoherent mixture, within a minimally realist, epistemic interpretation of the quantum formalism.

The goal of this paper is to elaborate on and extend the results of [11], strengthening this central claim in a number of ways. In Section 2, the proposed interpretation is compared to other, similar ways to understand the formalism that have been proposed in the past, and it is argued to be consistent with them; additionally, its implications for quantum measurement are briefly summarized and stated more compactly (and perhaps more sharply) than they were in [11]. Section 3 illustrates these principles by applying them to a large variety of possible measurement schemes. Section 4 makes a case for the reality of irreversibility in physics, and, in particular, characterizes the kind of irreversibility required to support the conclusions of the paper. The latter are then summarized in Section 5.

## 2. The Quantum Formalism and “Elements of Reality”

### 2.1. Limited Realism Within an Overall Epistemic Interpretation

The interpretation of the quantum formalism proposed here is fundamentally epistemic, meaning that the formal “quantum state” is taken to be just a mathematical representation of the information the observer has about the system. An essential part of it is that, in many cases, the observer may not have the maximal information allowed by quantum mechanics needed to fully characterize a pure state: in those cases, the proper description of the system is by a mixed-state density operator. The density operator, then (rather than the state vector) is regarded as the basic tool of the theory, even when dealing with individual systems.

This is in contrast with “ontic” interpretations of the formalism, which assume that every system must have a wavefunction “by default” (a wavefunction being just a pure state, or “state vector,” written in a certain basis). In an epistemic interpretation, a state vector is not something a system “has” (the way, say, a classical particle automatically has a position and a momentum), but rather something that we assign to it, and only when the information that we have, and can empirically verify, warrants it. In such an interpretation, the notion of an “unknown pure state” is a contradiction in terms, except in the very special case in which it refers to a state that has been prepared by somebody but is unknown to somebody else (but note that, even in this case, the correct description of the situation from that other person’s perspective should not be a pure state, but a density matrix that reflects their ignorance).

On the other hand, the particular epistemic interpretation proposed here and in [11] is “minimally” realist, in that it allows that, when we have enough empirically verifiable information about a system to warrant a pure state description, we can speak of an objective element of reality that is associated with that system, in the specific context considered. This view of the quantum state is consistent with Heisenberg’s, who in his book *Physics and Philosphy* writes the following [12] about the density operator (which he calls the “probability function”):

“The probability function combines objective and subjective elements. It contains statements about possibilities or better tendencies (“potentia” in Aristotelian philosophy), and these statements are completely objective, they do not depend on any observer; and it contains statements about our knowledge of the system, which of course are subjective in so far as they may be different for different observers. In ideal cases the subjective element in the probability function may be practically negligible as compared with the objective one. The physicists then speak of a “pure case.””

It is also consistent with the point of view spoused by A. Peres in [13,14], where he basically identifies a pure state assignment with a definite preparation procedure. It is, moreover, also consistent with the Pusey–Barrett–Rudolph (PBR) theorem [15], despite the latter having sometimes been held to prove the “ontic” nature of the wavefunction. In actual fact, what PBR establish is that we can assign a distinct element of reality to any state we can actually prepare (and empirically verify). As the latter is just Peres’s criterion for a valid pure state description, we may conclude that it is legitimate to associate an element of reality with any system that we can legitimately describe by a pure state. The most natural choice for such elements of reality would be the eigenvalues of a complete set of commuting operators of which the state in question is an eigenstate, as these would automatically satisfy the Einstein–Podolsky–Rosen criterion of reality [16] adopted in [11]: “If a physical property can be repeatedly and reliably observed, without in any way disturbing a system, there is an element of reality associated with that property.”

Finally, as regards the Schrödinger equation, whether for a pure state

(1) $$\frac{d}{dt}|\psi \rangle =-\frac{i}{\hbar }H|\psi \rangle$$

or a density operator

(2) $$\frac{d}{dt}\rho =-\frac{i}{\hbar }\left[H,\rho \right]$$

it should be emphasized that its job is merely to propagate a probability distribution in between observations. As such, it requires that the system be “informationally closed” over the time interval considered, that is to say, that no information “leak out” (or in). It would be incorrect to say that the Schrödinger equation mandates that quantum information must be conserved, as if this was some law of physics; rather, the equation *assumes* that information is conserved, and only applies if that is the case.

### 2.2. Implications for Quantum Measurement

Any epistemic interpretation automatically takes care of the collapse “on paper” of the quantum state during a measurement, since, in this view, it is just an updating of the probability distribution, as new information becomes available. However, any interpretation that assigns, even if only under limited circumstances, elements of reality to quantum systems, has to face some form of the “measurement problem.” Specifically, one still, at some point, may have to account for the fact that a property that the initial system possessed, and which was incompatible with the property being measured, can no longer be associated to an element of reality, whereas the property being measured can.

It was argued in [11] that this can, in fact, be done consistently if one of the measurement steps involves an irreversible process that makes the relative phase information in a coherent superposition irretrievably lost. That is, the key (as already known from decoherence theory [17,18]) is decoherence, but this decoherence needs to be at least *probably* (ideally, with high probability) irreversible. Under those circumstances, and within the proposed epistemic interpretation of the formalism, the fundamental (ontic) objection that the underlying entanglement with the rest of the larger system prevents the subsystem from truly having any well-defined properties of its own can be dismissed, and (with the corresponding probability) an objective collapse to a definite outcome can be envisioned. Interestingly, the required irreversibility can already be found at the microscopic level; this was a central point in [11], and will be reinforced by the examples presented here.

The full argument to establish the need for irreversibility may be found in [11], but the essence of it is that, if the evolution that leads to a state that evolved from a coherent superposition, and “looks like” a coherent superposition, is not reversible, then, in fact, the alleged coherence of the final state cannot be empirically demonstrated, and, by the criterion of reality adopted above, one is not required to assign an element of reality to the “lost” phase information. In a way, this perspective turns the ontic argument against the collapse on its head: instead of accepting that the larger system is correctly described by a pure state and the measured system, therefore, by an “improper” mixture, the argument holds that it is the pure state assignment that is “improper,” and the collapsed density operator is, in fact, *the* proper description of the system, since the formalism is supposed to represent only the empirically verifiable information we possess about it.

An added strength of this interpretation is that it legitimizes the use of open-systems methods [19,20,21] to describe the collapse, since, under the assumption of true irreversibility, the operation of tracing over the environment is justified, from an epistemic perspective, as a way to correctly describe the evolution of the verifiable information we possess about the system. This was already argued, with an example, in [11], and will be shown again here, for a different example, in Appendix A. (As for the question of what qualifies as “true” irreversibility, this will be dealt with at some length in Section 4, but, in the meantime, the examples below should suffice to get an idea.)

The examples in the following Section illustrate the application of the principles put forward here to several different types of quantum measurement: a non-demolition version of the Stern–Gerlach experiment, a dissipative version closer to the original experiment, and a variant of the qubit readout considered in [11] that maps to an “interaction free” measurement. Many variants of these schemes have been considered before, in one form or another (see, e.g., [22]); the key difference here is the emphasis on the interpretational framework just discussed and on the irreversible processes involved. In particular, as noted above, a key consequence of the proposed interpretation is that certain “pure-state like” expressions that seem to follow from Schrödinger’s equation, but actually describe the result of an irreversible process, should not be regarded as legitimate pure-state assignments but must be replaced by mixed states that truly reflect the loss of information about the initial state. In Section 3.4, it is shown how this approach can be applied to the Schrödinger cat example, and in Section 3.5 it is applied to the tracks left by a charged particle in a cloud chamber, a question that was debated for a while in the early days of quantum mechanics.

## 3. Measurement Examples

### 3.1. Non-Demolition Stern–Gerlach Experiment

**Figure 1:**
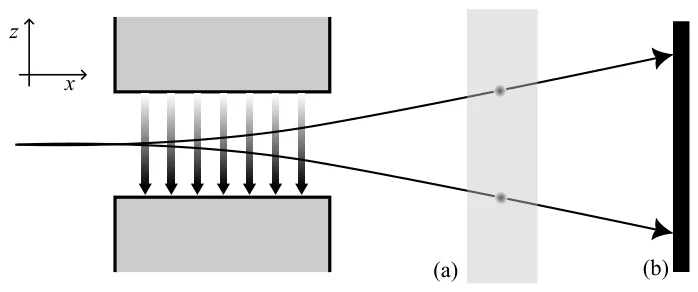
Two possible ways to carry out a Stern–Gerlach measurement on a spin-1/2 atom. (**a**) The atom passes through a region of space (lightly shaded in the figure) illuminated by a strong laser field tuned to its resonance frequency. The atom’s position is detected by observing the scattered light (fluorescence). (**b**) The atom instead collides with a glass plate at one of two possible locations. The “amplification” of the signal in this case results from sending a large number of atoms, which eventually produce a visible spot on the plate.

Consider a spin-$\frac{1}{2}$ particle (an atom, say) initially prepared in the spin state

(3) $${|\Psi \rangle }_{s}=\alpha |↑\rangle +\beta |↓\rangle$$

where |↑〉 and |↓〉 are the eigenstates of $S_{z}$. After passing through a Stern–Gerlach apparatus aligned along the *z* axis, as in Figure 1, the particle can follow one of two trajectories, given by the functions ${\vec{r}}_{+}\left(t\right)$ and ${\vec{r}}_{-}\left(t\right)$, depending on the direction of the spin. Although fluctuations in the magnetic fields and other environmental factors might cause some loss of coherence, for the purpose of the following discussion, it will be assumed that the experiment is carried out at atom-interference standards, and so the state coming out of the magnetic field region will be written as the coherent superposition

(4) $${|\Psi \rangle }_{sw}=\alpha |↑\rangle |\psi_{+}\left(t\right)\rangle +\beta |↓\rangle |\psi_{-}\left(t\right)\rangle$$

where the subscript sw refers to the system formed by the spin and center-of-mass wavefunction degrees of freedom. Ignoring, again for simplicity, wavefunction spreading, the functions $\psi_{\pm }\left(\vec{r},t\right)$ can be written as

(5) $$\psi_{\pm }\left(\vec{r},t\right)=\psi_{0}\left(\vec{r}-{\vec{r}}_{\pm }\left(t\right)\right)$$

where $\psi_{0}\left(\vec{r}\right)$ is an atom-size wavefunction, centered at $\vec{r}=0$.

Note that, at this point, no measurement has taken place yet. Equation (4), sometimes called the “pre-measurement” state, corresponds to the first stage in von Neumann’s description of an ideal measurement [23], in which the variable of interest (here, the spin) is coupled, via an entangling operation, to a probe (here, the particle’s center of mass), which is what will be observed subsequently.

The measurement postulate requires this observation to result in a state assignment that evolves as follows:

(6a) $$\begin{array}{cc} \; & |\Psi \rangle =\alpha |↑\rangle |\psi_{+}\left(t\right)\rangle +\beta |↓\rangle |\psi_{-}\left(t\right)\rangle \end{array}$$

(6b) $$\begin{array}{cc} \; & \to \rho_{\mathrm{coll}}={\left|\alpha \right|}^{2}\left|↑\rangle \langle ↑|⊗\right|\psi_{+}\rangle \langle \psi_{+}{\left|+|\beta \right|}^{2}\left|↓\rangle \langle ↓|⊗\right|\psi_{-}\rangle \langle \psi_{-}| \end{array}$$

(6c) $$\begin{array}{cc} \; & \to \;\mathrm{one}\;\mathrm{of}\;|↑\rangle |\psi_{+}\left(t\right)\rangle \;\mathrm{or}\;|↓\rangle |\psi_{-}\left(t\right)\rangle \end{array}$$

where the key issue is that it must be legitimate to interpret the “collapsed” density operator $\rho_{\mathrm{coll}}$ in (6b) to mean precisely what Equation (6c) says (the so-called “problem of definite outcomes”). From a realist point of view, this means that the atomic spin “loses” the objective property represented by the state vector α|↑〉+β|↓〉 and instead acquires either the property represented by |↑〉 or the one represented by |↓〉.

As suggested in Figure 1, a (conceptually) simple way to observe the position of the atom would be to, literally, shine light on it and look at the fluorescence. In practice, the light would, of course, have to be very intense (a laser beam), resonant or nearly resonant with an atomic transition and not disturb the spin orientation in the process; if the two spin orientations result in different transition frequencies, two beams, each tuned to one of the transitions, could, in principle, be used. Again, this is basically a thought experiment, so all irrelevant practical difficulties can be ignored.

What cannot be ignored, however, is an essential requirement on the wavelength of the light: if the position of the atom is to be resolved, the wavelength must be much smaller than the separation between the two trajectories. Calling Δz the average of the distance $|{\vec{r}}_{+}-{\vec{r}}_{-}|$ in the region of observation, we must have

(7) λ≪Δz

Now, as the atom scatters photons (through spontaneous emission), it receives a momentum “kick” of magnitude ℏk=2πℏ/λ, in a random direction, for every photon emitted. This results in a change to the wavefunction that can be written as

(8) $$\psi_{\pm }\left(t\right)\to ≃e^{i\vec{k}\cdot {\vec{r}}_{\pm }\left(t_{0}\right)}\psi_{\pm }\left(t\right)$$

if the emission takes place at the time $t_{0}$. This follows from the fact that the momentum displacement operator $\exp(i\vec{k}\cdot \hat{\vec{r}})$ can be approximated by $\exp(i\vec{k}\cdot \vec{r}\left(t_{0}\right))$ when acting on a wavefunction localized in a region much smaller than a wavelength around the point $\vec{r}\left(t_{0}\right)$. The result is a random phase factor on each of the terms in (4), which destroys the coherence of the linear superposition:

(9) $${|\Psi \left(t\right)\rangle }_{sw}=\alpha e^{i\phi_{+}}\left|↑\rangle \right|\psi_{+}\left(t\right)\rangle +\beta e^{i\phi_{-}}\left|↓\rangle \right|\psi_{-}\left(t\right)\rangle$$

with $\phi_{\pm }=\vec{k}\cdot {\vec{r}}_{\pm }\left(t_{0}\right)$. Note that, because Equation (7) requires k≫2π/Δz, the magnitude of the average relative phase, $\phi =\vec{k}\cdot \left({\vec{r}}_{+}\left(t_{0}\right)-r_{-}\left(t_{0}\right)\right)$, could easily exceed 2π. This means that even a single photon emission makes the relative phase of the two terms in (9) completely unpredictable. On an epistemic interpretation of the quantum state, this means that we should write the system’s state after the emission of the first photon (whether this photon is observed or not) as the density operator obtained by averaging ${|\Psi \left(t\right)\rangle }_{sw}\langle \Psi \left(t\right)|$ of Equation (9) over the relative phase ϕ to reflect our ignorance of this quantity. The result is the “collapsed” density operator, $\rho_{\mathrm{coll}}$, of Equation (6b):

(10) $$\begin{array}{cc} \rho & =\frac{1}{2\pi }\int_{0}^{2\pi }{|\Psi \left(t\right)\rangle }_{sw}\langle \Psi \left(t\right)|\;d\phi \\ \; & ={\left|\alpha \right|}^{2}\left|↑\rangle \langle ↑|⊗\right|\psi_{+}\rangle \langle \psi_{+}{\left|+|\beta \right|}^{2}\left|↓\rangle \langle ↓|⊗\right|\psi_{-}\rangle \langle \psi_{-}| \end{array}$$

At this point, it may seem that we have, at least, partly “solved” the measurement problem, since we have obtained the result (6b) from a perfectly legitimate argument that might just as well have been used by Bohr [24] or Heisenberg (in fact, the result follows directly from the famous “Heisenberg microscope” thought experiment in Section II.2 of [25]). However, note that we have not actually used the Schrödinger equation (for a closed system) to do so. In fact, the derivation above may be regarded as a “hidden” application of open quantum system methods, either through an unraveling of a master equation using stochastic trajectories, or invoking the action of Heisenberg–Langevin-type random “forces.”

If we had instead tried to treat the spin and the whole “measurement apparatus” (in this case, the atom and the scattered photons) as a closed system evolving under the Schrödinger Equation (1), the total state of the larger system after scattering one photon would have taken the form

(11) $${|\Psi \rangle }_{swp}=\alpha |↑\rangle |\psi_{+}\rangle |1_{+}\rangle +\beta |↓\rangle |\psi_{-}\rangle |1_{-}\rangle$$

where the states $|1_{\pm }\rangle$ represent a photon emitted from ${\vec{r}}_{\pm }$, respectively. Because of our assumptions (formally, the condition (7)) that these two photon states be distinguishable, tracing ${|\Psi \rangle }_{swp}\langle \Psi |$ over the field does yield again the result (6b); however, the combined SWP (spin-center of mass wavefunction–photon) state (11) has the form of a *coherent* superposition, and as such, according to the ontic interpretation, it would not be legitimate to conclude that it represents a situation in which one of the possibilities $\left|↑\rangle \right|\psi_{+}\rangle |1_{+}\rangle$ or $\left|↓\rangle \right|\psi_{-}\rangle |1_{-}\rangle$ is actually realized.

This difficulty follows, in part, from our common, “everyday” understanding of coherent superpositions: for instance, we all know that the state $(|↑\rangle +\left|↓\rangle \right)/\sqrt{2}$ does not represent a situation in which a spin “is” either polarized along the +z or the −z direction, but rather, it is the very different situation where the spin is definitely polarized along the +x direction. This understanding is captured by the PBR theorem, which establishes that the three states $(|a\rangle +\left|b\rangle \right)/\sqrt{2}$, |a〉, and |b〉, all correspond to different (non-overlapping) elements of reality. Note that, *formally*, there is nothing wrong with Equation (11); the paper by Scully et al. [22] has many examples of similar superpositions resulting from the coherent interaction of the atom with quantum fields in optical, or microwave, cavities. Such interactions are, in principle, reversible, and the resulting states could thus, also in principle, be shown to correspond to distinct elements of reality.

On the other hand, physically, the state ${|\Psi \rangle }_{swp}$ is the result of a process (spontaneous emission) traditionally regarded as incoherent, or even coherence-destroying (see, e.g., [26,27]), and, mathematically, it was derived by applying an equation originally meant for closed systems to what is, essentially, an open system. It is, therefore, legitimate to ask whether the coherence of the superposition (11) can, in fact, be verified empirically. As noted in [11] for a similar situation, an immediate difficulty follows from the fact that the standard treatment of spontaneous emission (based, for example, on the Weisskopf-Wigner approximation [28]; see [29] for a simple derivation) assumes open boundary conditions, meaning that the photon escapes to infinity; this already would make the coherence of the superposition, which requires access to the three systems involved, unverifiable. This conclusion does not change if we allow for the (more likely) possibility that the photon may simply be absorbed in the walls of the laboratory, or detected by a photodetector (which must be part of the measurement’s experimental arrangement), since in that case it is also lost.

The only thing that might help to establish, empirically, the coherence of the superposition (11), and with it its validity as a state assignment with a distinct element of reality attached to it, would be the existence of a decay channel that could, eventually, bring the photon *coherently* back to the atom. Such channels do exist in the paper by Scully et al. quoted above, and may also be found in other arrangements, such as those in [30] or [31] (where they affect only, typically, a small fraction of the spontaneously emitted photons), but they are not present in the experimental arrangement proposed here. It is also worth keeping in mind, when comparing the proposed measurement scheme to these or any other hypothetical alternatives, that quantum “reality” is contextual: what can or cannot be considered “real” in a given circumstance—which is what we are trying to establish here—does depend on the whole experimental arrangement.

From the epistemic perspective adopted here, the paramount consideration is that the state assignment should reflect accurately all the information we have that is relevant to the given situation, and nothing more. For the SWP system, in free space or in an environment that only allows for incoherent detection or absorption of the photon, the state assignment (11) is therefore misleading, and should be replaced by the density operator

(12) $$\rho_{swp}={\left|\alpha \right|}^{2}\left|↑\rangle \langle ↑|⊗\right|\psi_{+}\rangle \langle \psi_{+}\left|⊗\right|1_{+}\rangle \langle 1_{+}{\left|+|\beta \right|}^{2}\left|↓\rangle \langle ↓|⊗\right|\psi_{-}\rangle \langle \psi_{-}\left|⊗\right|1_{-}\rangle \langle 1_{-}|$$

This does express what we actually know, and can verify, about the photon, in the context given: namely, that it is “out there somewhere,” with a probability distribution that can be calculated from $|1_{+}\rangle$ or $|1_{-}\rangle$, correlated “classically” with either of the spin states |↑〉 and |↓〉, and the corresponding center of mass wavefunctions.

The result (12) can already, without the need for any further justification, be traced over the photon state to yield again Equation (6b), just as our former derivation in terms of random kicks. This result can, in turn, be given the interpretation (6c) (namely, the atom is definitely at one, and only one, of the positions ${\vec{r}}_{+}$ or ${\vec{r}}_{-}$), which will be confirmed by the fact that the subsequent fluorescence (which is only resolving a “classical” alternative) will always be detected as originating at only one of the two locations (see Section IV.C of [11] for a more in-depth discussion of this point).

### 3.2. Stern–Gerlach Experiment with Detection by a Screen

The presentation in the previous subsection made it clear that even a single photon emission can be said to cause the state collapse, but since that emission was caused by the driving by a large external field, which must result in the emission of a large number of photons, one may still get the impression that the collapse part and the amplification part of the measurement process are somehow inseparable. To the contrary, the detection method presented in this subsection (which is actually closer to the original Stern–Gerlach experiment [32]; see [33] for an English translation) shows clearly that the collapse and the amplification processes can be completely separated.

In the original SG experiment, the silver atoms struck a glass plate and “condensed” on its surface, and the macroscopic signal was just the result of the accumulation of a macroscopic number of atoms (see Figure 1b). However, for each and every one of those atoms, the collision with the screen represented an irreversible process: namely, an inelastic collision, where the atom’s kinetic energy and momentum were both dissipated into the glass plate.

A “toy” model for the process is developed and solved in the Appendix. It involves the atom being “trapped” in an adsorption potential well near the surface (resulting from the balance between the long-range van der Waals attraction and the short-range Pauli repulsion), where it oscillates and eventually loses its translational energy by coupling to the large number of degrees of freedom (phonons) of the solid. Assuming that the atom sees different phonon environments at the two possible strike points ($z_{+}$ and $z_{-}$), the master equation treatment in Appendix A shows that this damping process again results in a reduced density matrix for the atom, of the form

(13) $$\rho ={\left|\alpha \right|}^{2}\left|↑\rangle \langle ↑\right|⊗\rho^{++}+{\left|\beta \right|}^{2}\left|↓\rangle \langle ↓\right|⊗\rho^{--}$$

(This assumes that the spin orientation survives the collision, which in the case of an electronic spin is unlikely, but that is ultimately irrelevant, as this was never intended to be a non-demolition measurement.)

Of course, the underlying physics is simply that, depending on its location at the time of impact, the atom has delivered its energy to one of two orthogonal sets of modes of the plate. In an ontic interpretation of the formalism, committed to the idea that every system must, by default, “have” a pure state, one might want to express this by writing for the overall system, including the phonon environment, the state

(14) $$|\Psi \rangle =\alpha |↑\rangle |\psi_{+}\rangle |e_{+}\rangle +\beta |↓\rangle |\psi_{-}\rangle |e_{-}\rangle$$

where $|e_{\pm }\rangle$ are orthogonal “environment states,” so tracing over them yields the result (6b). However, in this case the justification for the pure state assignment (14) is even more questionable, from an epistemic viewpoint, than it may have been for Equation (11) in the previous subsection. Besides having no criterion to determine a relative phase in (14), we do not even know what the states $|e_{\pm }\rangle$ actually are. Under those conditions, the only proper representation possible of what we actually know about the system is that given by the density matrix (13), perhaps supplemented by the observation that, right after the impact, there will be a microscopic amount of energy deposited in the plate, either in the neighborhood of $z_{+}$ or $z_{-}$, but in any case transient and only classically correlated with the atom’s final position.

Note that the derivation of the result (13) in Appendix A does not make use of the “macroscopic” nature of the glass plate, nor does it at any point treat it “classically.” The key assumption is that the (quantized) energy reservoirs at $z_{+}$ and $z_{-}$ are independent. The single large “plate” could be replaced by just two small, separate pieces at the expected impact locations. Although they would still have to be “large” by atomic standards (i.e., consist of enough atoms to support a continuum, or near-continuum, of phonon modes, in thermal equilibrium), the number of *phonons* involved could be very small; that is, the energy actually dissipated in this irreversible process is still firmly in the quantum domain (for example, an estimate in [34] for a sticking collision of gold atoms, effusing from a furnace at 1670 K, with a copper surface at 300 K, puts the number of phonons involved at “at least six.”)

### 3.3. Interaction-Free Measurements

**Figure 2:**
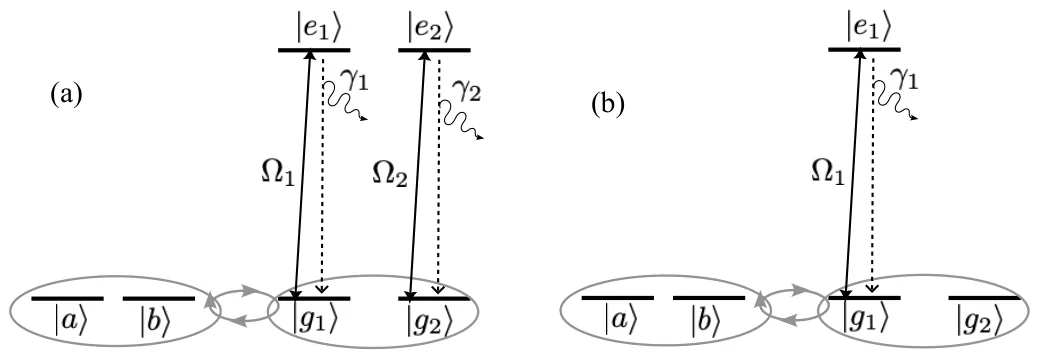
Two schemes for measuring a qubit. In case (**a**), discussed at length in [11], the probe (an atom) is observed by shining two laser beams, each resonant with one of the two possible transitions. In case (**b**), only one laser beam, resonant with transition 1, is used. In conventional language, the absence of fluorescence would “cause” the qubit’s state to “collapse” to |b〉.

In [11], a variation of a measurement scheme used for quantum information processing with atomic systems [35] was considered, as shown in Figure 2a below:

The system to be measured is a qubit, with basis states |a〉 and |b〉. The “probe” is an atom (or ion) with two degenerate ground states, $|g_{1}\rangle$ and $|g_{2}\rangle$, with which it is first entangled to produce the pre-measurement state $\alpha |a\rangle |g_{1}\rangle +\beta |b\rangle |g_{2}\rangle$. In case (a), two strong coherent fields are applied to the atom to take $|g_{1}\rangle$ and $|g_{2}\rangle$ to the excited states $|e_{1}\rangle$ and $|e_{2}\rangle$, respectively, and the resulting fluorescence (spontaneous emission) is monitored to determine whether the qubit’s state is |a〉 or |b〉, under the assumption that each state $|e_{i}\rangle$ can only decay to $|g_{i}\rangle$. It was argued in [11] that, independently of whether the fluorescence was monitored or not, the first spontaneously emitted photon on either transition would, even if undetected, suffice to warrant the reduction of the atom-qubit state to the “collapsed” form

(15) $$\rho =\rho_{\mathrm{coll}}={\left|\alpha \right|}^{2}\left|a\rangle \langle a|⊗\right|g_{1}\rangle \langle g_{1}{\left|+|\beta \right|}^{2}\left|b\rangle \langle b|⊗\right|g_{2}\rangle \langle g_{2}|$$

In practice, this measurement scheme can be more economically realized with only one transition being driven, say $|g_{1}\rangle \to |e_{1}\rangle$, as in Figure 2b. In this case, the *absence* of fluorescence would determine the system’s state to be $\left|b\rangle \right|g_{2}\rangle$. In fact, this is an example of what is called an “interaction-free measurement” [36,37,38] (see [39] for an English description of [36]), where the fluorescence in the driven transition plays the role of the “bomb” in [38]: if the bomb does not explode, we know that the particle went the other way. More precisely, and less metaphorically, if we ignore for a moment the fact that the atom is entangled with the qubit, this could be a perfect example of the system considered by Dicke in [37], where a wavefunction, which here would correspond to the atomic state $\alpha |g_{1}\rangle +\beta |g_{2}\rangle$, is “reduced” by shining on it light that only interacts with part of it—here represented by $|g_{1}\rangle$.

Compared with the case considered in Section 3.1, it is now not possible to blame “random kicks” for the collapse, since the center of mass wavefunction plays no role in the problem (the two states correspond to a single atom, at a fixed location in space, perhaps held in an optical trap). Also, unlike the case considered in Section 3.2, the collapse does not *necessarily* leave a trace in the environment, since no photons are emitted if the final state turns out to be $|g_{2}\rangle$.

As was done in [11] for the scheme in Figure 2a, it is helpful to consider first the simpler scenario in which only a finite pulse is applied to the transition $|g_{1}\rangle \to |e_{1}\rangle$, just long and strong enough to ensure that the state $|e_{1}\rangle$ would be reached if the initial state were $|g_{1}\rangle$ (a π pulse). Note that, if the lifetime of the state $|e_{1}\rangle$ is τ, the pulse itself needs to be much shorter than τ, in order to ensure an excitation probability close to 1. Immediately after the pulse, therefore, the state of the system, including the environment (in this case, the electromagnetic vacuum, represented by the state |0〉) is

(16) $$|\psi \rangle =\left(\alpha |a\rangle |e_{1}\rangle +\beta |b\rangle |g_{2}\rangle \right)|0\rangle$$

After a time of the order of (a few times) τ, it seems that we could describe the whole system in the “pure state” form

(17) $$|\psi \rangle =\alpha |a\rangle |g_{1}\rangle |1\rangle +\beta |b\rangle |g_{2}\rangle |0\rangle$$

where, as in Equation (11), an explicit expression for the single-photon state |1〉 could be obtained from the Weisskopf–Wigner (WW) approximation.

In Section 3.1, the argument was made that, in order to verify the coherence implied by the expression (11), it was necessary to “catch” the photon. Here, however, the very existence of the photon is uncertain! In fact, observing the coherence of a superposition state of the vacuum and one photon is in itself a rather tricky problem, and a state like (17) may be regarded as entangled by some criteria and not by others [40]. Nevertheless, the fundamental argument given in Section 3.1 remains valid: any attempt to verify the coherence of the superposition (17) would require a setup to bring the photon back, deterministically and coherently, and this is incompatible with the assumed free-space decay. Accordingly, a more truthful expression of what we can regard as “real” in this situation (that is, what we can empirically verify) would be given, in analogy to (12), by the density operator

(18) $$\rho_{\mathrm{coll}}={\left|\alpha \right|}^{2}\left|a\rangle \langle a|⊗\right|g_{1}\rangle \langle g_{1}{\left|⊗|1\rangle \langle 1|+|\beta \right|}^{2}\left|b\rangle \langle b|⊗\right|g_{2}\rangle \langle g_{2}\left|⊗|0\rangle \langle 0\right|$$

which simply says that the atom–qubit system may be in (i.e., have the properties described by the state) $\left|a\rangle \right|g_{1}\rangle$, in which case there is also a photon out there somewhere, traveling away from the atom, or it may be in the state $\left|b\rangle \right|g_{2}\rangle$, in which case there would be no such photon.

That being the case, we can now return to the initial scenario where the transition $|g_{1}\rangle \to |e_{1}\rangle$ is continuously driven and the fluorescence monitored by a detector aimed at the atom and covering a small enough solid angle, ΔΩ, to have a negligible effect on the atom’s decay rate. The probability to detect an emitted photon would be of the order of ΔΩ/4π, so, if after a time large compared to 4πτ/ΔΩ no photons have been detected, one can conclude that the state of the atom-qubit system is $\left|b\rangle \right|g_{2}\rangle$. It is worth noting that, as in all the other examples considered here, the time it takes for this “epistemic collapse” to happen (that is to say, for the observer to know what state to assign to the system) can be much longer than the time it takes for the system to lose its coherence, again an indication that the irreversibility and amplification parts of the measurement can be arbitrarily separated.

As to the “mechanism” behind the coherence loss in this example, it is to be noted that, unlike in the previous two cases, here there is, as Bohr once put it [41], “no question of a mechanical disturbance of the system under investigation.” Rather, it seems clear that just the *possibility* of spontaneous emission—that is, the availability of an irreversible decay channel—is enough to destroy the coherence of the initial state (16), after a time of the order of τ. As with other examples of “interaction-free measurements,” this is a dramatic illustration of Bohr’s contention that “there is still the question of an influence on the very conditions which define the possible types of predictions regarding the future behavior of the system.” Today, this is captured by the notion of contextuality: what can or cannot be considered “real” in quantum mechanics depends on the whole (experimental) context [42]. In the context described here, properties exhibited by coherent superpositions like $\alpha |a\rangle |g_{1}\rangle +\beta |b\rangle |g_{2}\rangle$, with α,β≠0, “cease to be real” after a time of the order of τ, whereas the properties associated separately with the states |a〉 and |b〉 are preserved, if present from the start—or can, presumably, become “real” once the incompatible properties associated with the coherent superpositions are gone.

### 3.4. Summary of Results; Schrödinger’s Cat

All the examples above make clear that the collapse is not due to the large physical size of some detector, which would, according to decoherence theory [18], prevent it from existing for more than an infinitesimal time in a superposition of macroscopically distinct states. On the contrary, the collapse happens on a microscopic scale, involving just an atom and a photon (in the examples in Section 3.1 and Section 3.3), or an atom and at most a few phonons, in Section 3.2. Although the part of the measurement apparatus responsible for the amplification of the signal does need to be suitably macroscopic, the part involved in the actual collapse (in these examples, the probe and the environment) does not have to be large, in the sense of having a large energy or a large number of particles, but only in the sense of offering a large (“infinite”) number of possible decay channels.

Noticeably absent also, in these examples, are the hypothetical von Neumann (or Wigner’s friends) chains of progressive entangled observers observing one another “coherently,” all the way up to some equally hypothetical “Heisenberg cut” (or to the consciousness of the last observer). Again to the contrary, the examples show that, in real-life measurements, decoherence occurs early, at the level of the “probe” itself (here always a single atom), and what gets amplified later can be legitimately described already by an incoherent (i.e., classical) sum of probabilities. (This is shown in a particularly clear way in Drossel and Ellis’s very detailed analysis of a photodetector [7].)

On the other hand, the examples do validate a crucial insight from decoherence theory [18], namely, the notion of “pointer states.” In all the cases, a system with states |a〉 and |b〉 becomes entangled with a probe with states |A〉 and |B〉, which is then made to interact with an environment in a way that preserves the states |A〉 and |B〉 themselves (or, more generally, the separate manifolds to which each of them evolve), but destroys the relative phase of their coherent superpositions. This makes |A〉 and |B〉 (or their corresponding manifolds) suitable “pointer states” for the measurement considered.

In all the examples, it was also crucial to treat the whole system quantum mechanically in order for the irreversible nature of the collapse to be apparent (it would not have done, for instance, to treat the screen in Section 3.2 as a purely classical object, unaffected by the absorption of the kinetic energy of a single atom). The examples also show that this can be done by standard methods of open quantum systems theory, whether master equations, as in the Appendices here and in [11], or the Heisenberg–Langevin or quantum trajectory unraveling methods hinted at in Section 3.1. Assuming true irreversibility, all of these are (always in an epistemic interpretation) legitimate ways to describe the way information about the initial state is partly lost in a quantum measurement.

On the other hand, “pure state” approaches based only on the Schrödinger equation and a closed-system assumption can lead to expressions, such as (11) or (17), that are misleading and need to be properly reinterpreted, since they look like coherent superpositions but cannot be regarded as legitimate pure state assignments in a truly irreversible process. Essentially, any state that represents the outcome of an irreversible process should be treated as introducing a random phase, to be averaged over so as to yield an appropriate incoherent mixture, as in Equation (12) or (18); this is because, in the absence of a coherent “return” channel, the original relative phases between that term and the others in the superposition become empirically inaccessible.

As a somewhat facetious example, it may be worthwhile to consider what such a rule would yield when applied to Schrödinger’s famous example [43] of a cat in a box with a radioactive sample, a Geiger counter, and a sealed glass bottle containing cyanide gas, to be broken when the counter clicks (see Section I.11 of [2] for an English translation). One might possibly write something like the following, for the state of the system at a time when it is not certain whether the sample has decayed or not:

(19) $$|\Psi \rangle ={\alpha |e\rangle }_{s}{|nf\rangle }_{gc}{|nb\rangle }_{b}{|nd\rangle }_{g}{|a\rangle }_{c}+{\beta |g\rangle }_{s}{|f\rangle }_{c}{|b\rangle }_{b}{|d\rangle }_{g}{|d\rangle }_{c}$$

(where the subscripts mean, in order, sample, geiger counter, bottle, gas, and cat, and the contents of the respective kets stand for excited/ground, not fired/fired, not broken/broken, not dispersed/dispersed, and alive/dead). This is done here, however, only to show explicitly that Schrödinger has piled up not just one or two, but five irreversible processes: the radioactive decay, the firing of the counter, the breaking of the glass, the dispersal of the gas, and the death of the cat. Even just one of them would suffice, according to the rule above, to force the replacement of (19) by the completely separable expression

(20) $$\begin{array}{cc} \rho = & {\left|\alpha \right|}^{2}{|e\rangle }_{s}\langle e|⊗\rho^{\left(gc\right)}\left[nf\right]⊗\rho^{\left(b\right)}\left[nb\right]⊗\rho^{\left(g\right)}\left[nd\right]⊗\rho^{\left(c\right)}\left[a\right]\\ \; & +{\left|\beta \right|}^{2}{|g\rangle }_{s}\langle g|⊗\rho^{\left(gc\right)}\left[f\right]⊗\rho^{\left(b\right)}\left[b\right]⊗\rho^{\left(g\right)}\left[d\right]⊗\rho^{\left(c\right)}\left[d\right] \end{array}$$

in terms of hypothetical density matrices, whose role is simply to suggest that, in principle, at least some aspects of the relevant states of the corresponding objects could be amenable to a quantum-mechanical description (e.g., the thermodynamics of the gas), but the statistical superposition, and the correlation it implies, is purely classical. Basically, Schrödinger could not have chosen a better example of a macroscopic system that could not be described by a coherent superposition—except that the ultimate reason for this is *not* the macroscopic size of the objects, but the irreversibility of the processes involved.

### 3.5. Cloud-Chamber Tracks

Finally, it is of interest to consider how the point of view adopted here provides a way to understand a problem that was the subject of some debate in the early days of quantum mechanics, namely, the “collapse of the wavefunction” represented by the visible tracks left by charged particles in cloud chambers.

In a 1929 paper [44], C. G. Darwin raised the question of how a “wave theory” could account for the straight line tracks observed, for instance, when an α emitter is placed in a cloud chamber, if one takes the initial state of the wavefunction representing the emitted α particle to be a spherical wave. Darwin conjectured that, if one could, in fact, solve the joint Schrödinger equation for the α particle and all the scatterers in the chamber, the result would indeed display straight-line motion for the α particle, but only in what we would call today a massively entangled, coherent superposition of all the possible straight-line rays from the origin (and all the corresponding scatterers’ states). He then argued that such a result would show that there was no need to think of the individual scattering events as “collapsing” the wavefunction as each of them was observed, but that the observation could be postponed until all the events were over, at which point the whole state would “collapse” into a straight line trajectory. (The paper is obviously remarkable in the way it seems to anticipate many later works, such as consistent histories and the many-worlds interpretation, in addition suggesting a possible role for the consciousness of the observer in the state reduction process.)

**Figure 3:**
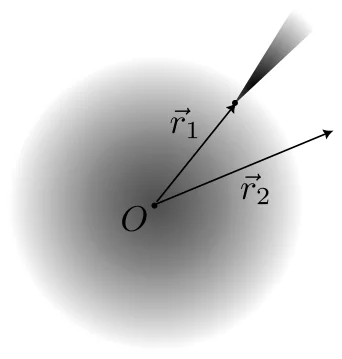
Illustrating Mott’s result. If the initial wavefunction of the α particle is a spherical wave centered at *O*, the probability that it will first excite an atom at ${\vec{r}}_{1}$ and then a second one at ${\vec{r}}_{2}$ is negligible unless the vectors ${\vec{r}}_{1}$ and ${\vec{r}}_{2}$ are nearly collinear, that is, unless the point ${\vec{r}}_{2}$ lies within the narrow cone originating at the point ${\vec{r}}_{1}$ shown in the figure.

Darwin’s conjecture was soon proved, in part, by Mott [45], who showed, by integrating the Schrödinger equation for three particles, that the joint probability of the α particle exciting an atom at ${\vec{r}}_{1}$ and then another one at ${\vec{r}}_{2}$ is very small unless the vectors ${\vec{r}}_{1}$ and ${\vec{r}}_{2}$ are collinear (with the center of the original spherical wave at the origin of coordinates; see Figure 3).

Mott chose not address the thorny question of when the collapse took place, but his construction has been taken to support Darwin’s contention that it could be arbitrarily postponed, since it does not make use of the collapse postulate at the location of the first scatterer. Interestingly, scarcely one year later, Heisenberg dealt with the problem in [25] (Section V.1), and presented his own version of Mott’s calculation, but his point was basically that it does not matter whether you treat the scatterers as part of the dynamical system being investigated, or as part of the observation apparatus, or even whether you treat the α particle quantum mechanically at all (!).

However, the point of view presented here does offer a definite answer, because an atom excited by the α particle must decay by spontaneous emission, or by ionization, and both are irreversible processes. This means that, after the first scattering event, the system’s state, instead of a coherent superposition involving all the positions of the possible scatterers,

(21) $$|\Psi \rangle =\sum_{i=N}^{N}C_{i}|\psi_{i}\left(\vec{r},{\vec{r}}_{i}\right)\rangle$$

should be represented as the incoherent superposition

(22) $$\rho =\sum_{i=N}^{N}|C_{i}{|}^{2}|\psi_{i}\left(\vec{r},{\vec{r}}_{i}\right)\rangle \langle \psi_{i}\left(\vec{r},{\vec{r}}_{i}\right)|$$

where each of the $\psi_{i}\left(\vec{r},{\vec{r}}_{i}\right)$ is a function originating in the neighborhood of the point ${\vec{r}}_{i}$ and extending over a narrow cone around the direction of the corresponding vector ${\vec{r}}_{1}$. Thus, as in the previous examples in this section, the first, microscopic, irreversible event collapses the state and determines the course of subsequent events (although in this case one does have a chain of successive collapses, happening at random places along the particle’s trajectory, but always approximately along a straight line).

Note that, also as in the previous examples, the amplification process by which we actually see the trajectory is separate from the collapse, and, in this case, involves a completely different (but also irreversible) physical process, namely, the condensation of a droplet of water around the position of the ion. Again, these condensation events can be regarded as merely displaying a track of ions that has already “objectively” been laid down.

## 4. Irreversibility

### 4.1. The General Case for Irreversibility

Although quantum measurement has been described as an irreversible act of amplification (e.g., by Wheeler in [2], paraphrasing Bohr in [24]), very few studies appear to have taken the requirement of irreversibility seriously. One of these is [46], where A. Peres argues that a true measurement requires irreversibility and proposes ways in which quantum mechanical systems could exhibit something equivalent to the kind of “irreversibility” encountered in the chaotic systems of classical nonlinear mechanics. In the same year, the notion that natural physical processes (in particular, spontaneous emission) might provide the irreversibility needed in a measurement was brought up briefly by van Kampen [47], who even observed that the key requirement was not the physical size of the “apparatus” but the coupling to an infinite number of decay channels. His paper, however, failed to provide a consistent interpretation of the quantum formalism in which this insight could have been properly embedded. Only much later did Drossel and Ellis [7] show, with a detailed quantum description of a measurement apparatus (a photodetector), that thermodynamic irreversibility at the microscopic level was all that was required to justify the collapse postulate, although their ontic interpretation of the wavefunction and their belief in strong emergence led them to see this result as setting a limit to the validity of what they called “pure” quantum mechanics.

Questions of interpretation aside, a distinctive advantage of Drossel and Ellis’s work is that they take irreversibility seriously, as a real feature of the universe, and not just a consequence of coarse-graining or some such thing. In fact, their paper lists as many as four “local” arrows of time, all ultimately related to the “cosmological” one. In this paper, as in [11], I have made extensive use of the radiation arrow, which is both straightforward and ubiquitous. The standard reason people give to dismiss irreversibility as only “apparent” is that the basic equations of physics, at the fundamental particle level, are all formally time-reversible (at worst, in combination with other symmetries, in very special cases like the kaon decay). While this is true, it ignores the fact that the actual solutions to the equations may well break this symmetry, depending on the boundary conditions.

Any two interacting charged particles will accelerate, and, as a result of that, emit electromagnetic radiation. The wave equation is certainly time-symmetric, but under normal boundary conditions, the emitted radiation will break that symmetry by taking the form of an outgoing wave, which carries away both energy and information about the initial state of the particles. All material objects contain charges, and all will radiate, at any temperature other than absolute zero. A rough estimate finds that the Earth radiates out to space some $10^{22}$ thermal photons per m^2^, per second, as part of its energy/entropy balance (assuming Planck’s law and an effective blackbody radiation temperature of 255 K at the “top of the atmosphere;” see, e.g., [48]). By any definition of information, classical or quantum, each one of those photons is carrying information about the process that produced it (and, by extension, about the whole history behind it). It also appears extremely unlike that any of them might come back, in any finite time. As people trying to build quantum computers know only too well, there is nowhere on Earth where we could draw an imaginary box and say “everything inside this box is a perfectly closed system, about which no information ever leaks out” [49].

(As an aside, given that we live on a planet where life as we know it would, literally, be impossible without dissipation [50,51], it seems that, from any position rooted in empiricism, the burden of proof should be on anybody who wishes to argue *against* irreversibility, rather than the other way around. In the context of quantum measurement, for example, when someone talks about reversing the state of Wigner’s friend’s mind, or assumes the possibility of perfectly isolating a system from its environment for an unspecified length of time, asking how this might be physically achieved should not be regarded as an impertinence; it would be, on the contrary, a very pertinent, scientific question.)

### 4.2. The Irreversibility Needed for Quantum Measurements

In all the measurement examples considered in this paper, the loss of the crucial phase information needed to establish, empirically, the reality of an alleged coherent superposition rests on the above “no return” assumption, for a particle or quasiparticle leaving the system. Note that, unlike in the classical case, reversibility in these examples requires not only that the particle return, but that it return “coherently.” For instance, in the case considered in Section 3.2, the energy lost by the atom should not be delivered back at one of the two possible impact locations, but in some sort of superposition of both so that it would not be possible to tell where the atom hit the screen. Keeping in mind that the measurement is supposed to take place in a context where no special effort is made to set up such a coherent return channel, it is hard to imagine how this could take place with anything other than a vanishing small probability in any of the examples considered here.

One could then formally characterize the kind of irreversible process required for a quantum measurement as a spontaneously occurring process, in which one or more information-carrying particles or quasiparticles leave the system, without a coherent return channel. This means, among other things, that, if they are monitored at all, it is not in any way that would allow their being coherently returned to the system being measured. Note that this can describe many different processes, in addition to the ones considered in Section 3: for instance, any process that ultimately results in the emission of thermal photons, as mentioned above, including exothermic chemical reactions, inelastic molecular collisions, and, ultimately, any kind of thermalization process, such as the one considered by Drossel and Ellis.

Mathematically, these processes should naturally be described by open boundary conditions, which typically include integrals over a continuous infinity of modes that automatically preclude recurrences. Although this involves, potentially, an infinite number of degrees of freedom, our examples show that some of these processes, involving small numbers of particles, can even be handled, with minor approximations, by the closed-system Schrödinger Equation (1). In that case, the interpretation of the final result plays a critical role. In the epistemic interpretation, the quantum state carries, by itself, no ontic weight at all, and it is only meant to keep track for us of the available information. In that case, as has been argued above, the correct procedure is to replace a state that looks like a coherent superposition, but whose coherence, because of the “no return” condition, could never be empirically verified, by an appropriate incoherent mixture.

Alternatively, of course, one can simply trace over the unobservable degrees of freedom, which is what is routinely done in the open quantum system methods that have been used successfully to describe irreversible quantum dynamics for a very long time now. Either way, the key observation is that there are, in fact, a multitude of naturally occurring processes that exhibit the same dynamics that causes the “collapse” or sate reduction, and a well-designed quantum measurement simply makes use of some of them. None of these processes require for their description any “new” dynamics not contained in the Schrödinger or Heisenberg equations, only the proper boundary conditions. All of them cause decoherence, and all of them are physically irreversible (regardless of how deceptively reversible the mathematical results may appear); nevertheless, when they are used in the context of a measurement, an appropriate interpretational framework is still required to justify the collapse postulate.

Finally, one more thing that should perhaps be mentioned in a discussion of irreversibility and its relevance to quantum measurement is that all *classical* measuring devices must also rely on irreversible processes to produce a “permanent” record: scales rely on friction, electronic devices on resistors, etc. Dissipation, in fact, is essential for defining a device’s response time. This may be a reason why none of the theory’s founders ever tried to describe a “classical” measuring apparatus quantum mechanically, since they could not expect their unitary evolution formalism to be applicable to such an object in the first place. Ironically, however, the results presented here indicate that the quantum “state collapse” is not due, directly, to this “higher level” irreversibility, but rather to irreversible microscopic processes that take place prior to, and largely independently of, the amplification stage.

## 5. Conclusions

The main result from this work is a challenge to two very commonly accepted notions in quantum measurement theory. The first one, historically, is that of an entangled “von Neumann chain” of measuring instruments measuring each other, none of them in a definite state; the second one is the notion that the “break” in the chain (if indeed there is one) is ultimately due to the “classical” nature of the measurement apparatus. A result often mentioned, in this context, is the typically very fast decay of the coherences between macroscopically distinct states, which would render them “for all practical purposes” unobservable.

By contrast, it has been argued here that the von Neumann chain is purely speculative and does not need to describe real quantum measurements. Rather, in typical examples, the collapse happens already at the *quantum* level of the system and the probe (here, single atoms), interacting with environments that are either vacua or contain very few excitations at the relevant energies. The key physical requirement is the irreversibility of the probe-environment interaction; the result then follows consistently from a fundamentally epistemic interpretation of the quantum formalism that only allows for the “reality” of certain quantum properties under limited and strictly controlled conditions.

With the chain thus broken at its very earliest links, the arguments for the collapse from macroscopic decoherence become redundant: in a properly designed quantum measurement, there must be, of course, an amplification process, but this, according to our interpretation, may never have to deal with coherent superpositions and only serves to bring to the attention of the observer a pre-existing definite outcome. This also makes arguments involving nonlinear dynamics or (classical) chaos irrelevant to the state reduction or collapse question. In all the examples presented here, everything relevant happens at the quantum level and can be described correctly by the methods of the theory of open quantum systems; the validity of these methods, which may appear dubious in an ontic interpretation (inasmuch as it involves tracing over the “environment” degrees of freedom), seems quite plausible from an epistemic viewpoint, since their job, from this perspective, is merely to describe the leakage of information, by incorporating as much as necessary of the joint system-environment quantum dynamics.

It is important to stress that, in essence, these methods are nothing but another form of decoherence theory, with the only difference from the way it is normally applied to the measurement problem being the physical size of the systems considered. The only substantial disagreement between this work and some previous ones that make use of decoherence theory to support a many-worlds interpretation is ontologic. The epistemic interpretation proposed here automatically accepts the existence of elements of reality associated with pointer states (since they trivially satisfy the EPR criterion) but does reject the existence of such elements of reality for the relative phases of the superposition states appearing in the typical von Neumann chain when their alleged coherence cannot be empirically verified, either because they result from an irreversible process like (11) and (17), or because they involve “unknown pure states” like (14). Once this is done, as has been shown here, an objective interpretation of the state collapse becomes possible, and any need for a “many worlds” interpretation of the formalism vanishes.

Similarly, it should also be noted that, while the proposal here fully agrees with Drossel and Ellis [7] on the essential role played by irreversibility in the measurement process, it also disagrees with their ontology: whereas they say that the wavefunction is real and that irreversibility cannot be described by “pure” quantum mechanics, the point of view adopted here is that the wavefunction is only a mathematical construct, and that the methods mentioned above to describe irreversible processes are as much a part of genuine quantum mechanics as anything else.

Finally, it goes without saying that (as is usually the case) nothing in the interpretation proposed here leads to predictions of experimentally verifiable discrepancies with any of the many other possible interpretations of quantum mechanics. The goal here and in [11] has been simply to show that there is, at least, one consistent, all-quantum account of the measurement process, including both state collapse and definite outcomes, that does not need to postulate infinite universes or completely forsake realism. It does require, however, “taking irreversibility seriously.”

## Data Availability

The original contributions presented in this study are included in the article. Further inquiries can be directed to the corresponding author.

## Appendix A

An early, classical treatment of the collision of an atom with a solid surface is due to Zwanzig [34], who modeled the surface as a semi-infinite, one-dimensional chain of oscillators. More sophisticated treatments have been developed over the years [52], including a Langevin equation approach in [53]. The basic idea behind most of these (including quantum scattering treatments [54]) is that the atom oscillates in a potential (often taken to be of the Morse form) close to the surface, while losing energy through its interaction with phonons in the solid.

The goal of this appendix is not to attempt an accurate description of any particular system but rather to illustrate the consequences, for the Stern–Gerlach measurement considered in Section 3.2, of a process like the one just mentioned using the simplest model possible. This will involve quantizing the motion of the atom in the *x* direction (perpendicular to the surface) and using a master equation that corresponds, essentially, to the Markovian limit of the Langevin equation in [53], with a simple harmonic potential. Introducing the creation and annihilation operators *a* and $a^{†}$,

(A1) $$\begin{array}{cc} a & =\sqrt{\frac{m\omega_{0}}{\hbar }}\left(x+i\frac{p_{x}}{m\hbar \omega_{0}}\right)\\ a^{†} & =\sqrt{\frac{m\omega_{0}}{\hbar }}\left(x-i\frac{p_{x}}{m\hbar \omega_{0}}\right) \end{array}$$

the Hamiltonian, in the interaction picture, involves the creation and annihilation of phonons in two separate reservoirs, corresponding to the two possible locations, $z_{+}$ and $z_{-}$, where the atom may hit the surface:

(A2) $$\begin{array}{cc} H= & \int g\left(\omega \right)\left(b_{+}\left(\omega \right)a^{†}e^{-i(\omega -\omega_{0})t}+H.c.\right)d\omega \;P_{+}\\ \; & +\int g\left(\omega \right)\left(b_{-}\left(\omega \right)a^{†}e^{-i(\omega -\omega_{0})t}+H.c.\right)d\omega \;P_{-} \end{array}$$

where the projectors $P_{\pm }$ ensure the interaction is restricted to the appropriate part of the wavefunction (formally, for instance, one could write $P_{+}=\left|↑\rangle \langle ↑\right|$, $P_{-}=\left|↓\rangle \langle ↓\right|$). This is a reasonable approximation if the separation between the impact locations is much larger than the interatomic distance; it does not even have to be an approximation, since one cannot expect the physics to change in any substantial way if the screen was actually split into an upper and a lower part, or even confined to small regions around $z_{+}$ and $z_{-}$. Note that, again to simplify the treatment, the rotating-wave approximation has been made in Equation (A2).

A master equation for ρ can then be obtained in the standard way (see, e.g., [20], Section 1.4.) by iterating Equation (2) twice and averaging over the reservoir modes, assuming that in the second-order term $\rho_{\mathrm{total}}\left(t\right)≃\rho_{\mathrm{sys}}\left(t\right)⊗\rho_{res}\left(0\right)$. Further assuming the coupling g(ω) between the atom and the phonons is a sufficiently flat function of the frequency, one gets the Markov approximation

(A3) $$\int g^{2}\left(\omega \right)e^{-i\omega (t-t^{\prime })}\;d\omega ≃2\pi g^{2}\left(\omega_{0}\right)\delta \left(t-t^{\prime }\right)$$

The final result takes the form

(A4) $$\begin{array}{cc} \dot{\rho }= & \gamma \left(\bar{n}+1\right)\left(2aP_{+}\rho P_{+}a^{†}+2aP_{-}\rho P_{-}a^{†}-a^{†}a\rho -\rho a^{†}a\right)\\ \; & +\gamma \bar{n}\left(2a^{†}P_{+}\rho P_{+}a+2a^{†}P_{-}\rho P_{-}a-aa^{†}\rho -\rho aa^{†}\right) \end{array}$$

with $\gamma =\pi g^{2}\left(\omega_{0}\right)$, and $\bar{n}\equiv \bar{n}\left(\omega_{0},T\right)$, where, if we assume the reservoir is in thermal equilibrium at a temperature *T*,

(A5) $$\bar{n}\left(\omega ,T\right)=\frac{e^{-\hbar \omega /k_{B}T}}{1-e^{-\hbar \omega /k_{B}T}}$$

In the pure decay terms of Equation (A4), the relation $P_{+}+P_{-}=1$ has been used. The same result can be used to write ρ as

(A6) $$\rho =\rho^{++}+\rho^{--}+\rho^{+-}+\rho^{-+}$$

where, for example, $\rho^{+-}=P_{+}\rho P_{-}=|↑\rangle \rho |↓\rangle$, etc. It may be worth emphasizing that we are not really assuming that the spin orientation survives the collision; here, as in the main text, we are essentially just using |↑〉 and |↓〉 as markers for the parts of the wavefunction associated with each of the two impact points.

In particular, $\rho^{+-}\left(t\right)$ and $\rho^{-+}\left(t\right)$ will give the evolution of the coherence initially present in the state (9). it is easy to see that, for an arbitrary matrix element,

(A7) $${\dot{\rho }}_{nm}^{+-}=-\gamma \left[m+n+2\left(n+m+1\right)\bar{n}\right]\rho_{nm}^{+-}$$

which shows that the coherence decays at least as fast as γ, and in general much faster, depending on the initial energy in both the system and the environment. On the other hand, $\rho^{++}$ and $\rho^{--}$ approach a state of thermal equilibrium with the phonon bath, with $\langle n\rangle =\bar{n}$.
